# Patients' Experiences With Advance Care Planning and Decision‐Making: An Interview Study in Finnish Hospital Palliative Care Wards

**DOI:** 10.1002/nop2.70158

**Published:** 2025-02-18

**Authors:** Anne Kuusisto, Kaija Saranto, Katriina Lähteenmäki, Anu Soikkeli‐Jalonen, Elina Haavisto

**Affiliations:** ^1^ Department of Nursing Science University of Turku Finland Turku Finland; ^2^ The Wellbeing Services County of Satakunta Pori Finland; ^3^ Department of Health and Social Management University of Eastern Finland Kuopio Finland; ^4^ Health Sciences Unit of the Faculty of Social Sciences Tampere University Tampere Finland

**Keywords:** advance care planning, advance directives, death, inter‐professional relations, nursing, palliative care, patient care planning, patients, qualitative research, shared decision‐making

## Abstract

**Aim and Objective:**

This study described patients' experiences with advance care planning and decision‐making in Finnish hospital palliative care wards.

**Design:**

A descriptive qualitative study with semi‐structured individual interviews.

**Methods:**

The study group consisted of purposely selected patients in palliative care wards from two university hospital districts. A pretested interview guide was used. The interviews focused on three main themes with auxiliary questions. Data were gathered until data saturation was reached. The data were analysed using inductive content analysis.

**Results:**

A total of 20 patients with cancer were interviewed. Patients' experiences with advance care planning in palliative care were grouped into three parent categories with subcategories: (1) Making plans for the end of life (need for psychosocial support in cancer disease and wish for goals of care discussion), (2) Symptom management planning (wish for pharmacological interventions for symptom management and wish for non‐pharmacological interventions for symptom management) and (3) Palliative care coordination (need for discharge planning and wish for compatibility between team members). Patients' experiences with care decision‐making in palliative care were grouped into two parent categories with subcategories: (1) healthcare professional as a care decision‐maker (medical care decision‐making, nursing care decision‐making and inter‐professional care decision‐making) and (2) shared decision‐making (need for patient involvement in shared decision‐making and need for family member involvement in shared decision‐making).

**Conclusion:**

This study highlights the need for advance care planning and involvement in shared decision‐making in palliative care from patient perspectives.

**Relevance for Clinical Practice:**

The results from this study show that nurses must be critically concerned about the early and intentional initiation of palliative care.

**Reporting Method:**

The consolidated criteria for reporting qualitative studies checklist (COREQ) was used.

**Patient or Public Contribution:**

The data consists of answers given by patients in interviews.

## 1 Introduction

Important aspects of care planning and decision‐making in palliative care are an individual approach, communication, and recognising dying (Queensland Health 2019). Patients in need of palliative care have specific needs concerning end‐of‐life care (Kowitt et al. 2019; Sharafi et al. 2022; Staats et al. 2023). They can, for example, receive care in different institutions during the disease continuum (Kaasa et al. 2018; van Doorne et al. 2021; Hui et al. 2023) and may often be hospitalised at the end of their lives (van der Padt‐Pruijsten et al. 2022; Ouchi et al. 2023). In addition, the development of modern oncology treatments has brought new types of care planning and decision‐making challenges into palliative care (Temel et al. 2022). Thus, end‐of‐life care planning with a patient‐centred care pathway (Kaasa et al. 2018) is important in terms of continuity of care (Zulueta Egea et al. 2023).

A systematic review by Coulter et al. (2015) shows that individual care planning means patients' active involvement in their care decision‐making. It includes goal setting and an action plan agreed upon through discussion, which supports the patient's overall service chain. Individual care planning with shared decision‐making improves adults' health and the ability to self‐manage long‐term health conditions compared to routine care. (Coulter et al. 2015.) Care planning is guided by ethics, legislation (Act on the Status and Rights of Patients 1992), guidelines (Institute of Medicine 2014; Queensland Health 2019), and culture (Kuusisto et al. 2023). According to recommendations, all individuals should have the possibility to take part in their care decision‐making at all stages of life. As they near the end of life, they should have access to medical and pertinent social services consistent with their goals, values and informed preferences (Institute of Medicine 2014).

Advance care planning (ACP) as a special part of care planning (Kuusisto et al. 2020; Burghout et al. 2023) focuses on the expected deterioration in patients' condition when nearing death (Kaasa et al. 2018). According to the definition produced by an international panel of experts, ACP “enables individuals to define goals and preferences for future medical treatment and care, to discuss these goals and preferences with family and health‐care providers, and to record and review these preferences if appropriate” (Rietjens et al. 2017). ACP (Starr et al. 2019) applies Shared Decision‐Making (SDM), which can be defined in many ways between patients, healthcare professionals, and families to recognise patients' goals and priorities for their end‐of‐life care (Gerber et al. 2019; Kuosmanen et al. 2021; Wang et al. 2023). ACP should occur as long as individuals are able to make decisions about their own care (Kim et al. 2023). However, ACP is underutilised (Beck et al. 2023; Goswami 2023; Guccione et al. 2023) and established practices are lacking (Kuusisto et al. 2020) despite the perceived benefits of ACP (Wang et al. 2023) and efforts to support Shared Decision‐Making (Baik et al. 2019; Bukstein et al. 2020; Volandes et al. 2023).

Good palliative care should be patient‐orientated and based on the patient's needs and views (Sjöberg et al. 2022; Zulueta Egea et al. 2023). Nonetheless, the goals of patients and healthcare professionals regarding care planning and decision‐making may not be consistent (Ellis et al. 2019). Studies from different countries are known to have significantly different cultural influences, particularly within the context of end‐of‐life care planning (Kuusisto et al. 2023; Zhu et al. 2023). This is specifically important to consider when comparing the readiness to talk about end‐of‐life topics. For example, in the Nordic culture in Finland, healthcare professionals often find end‐of‐life discussions with patients to be an uncomfortable topic. Only a few list their own wishes and dare to deal with the end of life in time. (Kuusisto et al. 2023) Thus, death is quite a private issue. To the best of our knowledge, there are no studies describing patients' experiences in palliative care regarding advance care planning and decision‐making in the same research in the Nordic context in Finland. From the point of view of developing palliative care, obtaining information on the topic is essential. Thus, this study aims to describe patients' experiences with advance care planning and decision‐making in Finnish hospital palliative care wards.

## 2 Methods

### 2.1 Study Design

A descriptive qualitative study was conducted with semi‐structured individual interviews to explore and understand the topic under research (Renjith et al. 2021). The consolidated criteria for reporting qualitative studies (COREQ) checklist was used to report all the main points related to the study (Tong et al. 2007) (Data S1).

### 2.2 Study Context

This study was performed in two of Finland's twenty hospital districts (as of 1 January 2023, known as Wellbeing Services Counties). These two large university hospital districts provide public specialised medical care and outpatient treatment for roughly 39% of the Finnish population. In Finland, palliative care is guided by several laws and recommendations. In the country, palliative care services are organised according to the three‐step organisation model of palliative care and end‐of‐life care at basic (A), special (B), and demanding special level (C) according to the level of care necessitated. At basic level A, palliative care is offered, along with other activities, in all social and health care units that care for patients at the end of their lives. At special level B, e.g., hospital wards specialise in palliative care and end‐of‐life care. At demanding special level C, university hospital palliative care centres organise special level care in local government co‐management areas. In this study, the data were gathered in four special‐level (B) hospital wards whose speciality is palliative care and where the personnel is specifically trained.

### 2.3 Study Participants and Data Collection

The study group consisted of purposely selected patients in hospital palliative care wards (Renjith et al. 2021). The sampling criteria were the following: patients over 18 years of age, Finnish‐speaking, had an incurable disease, received at least 1 week of hospital‐based palliative care, and were capable of giving opinions of their individual care experience. Each ward had a contact person who was responsible for recruiting interviewees and appraising each patient's suitability for the interview. Before the interviews, the contact persons gave the interviewees written information about the research goals and the interviewers' position in the research project. Moreover, the interviewees filled out a background information form, and their informed consent was ensured.

Two researchers (AS‐J, EH) collected the data in the spring and fall of 2019. The interviewers did not establish any relationship with the patients prior to study commencement. A pretested interview guide was used (Table 1). User involvement was used when the interview guide was developed. The interviews were focused on three main themes with auxiliary questions. The interviewees were asked to describe their individual experiences and give examples of the following aspects of their care: (1) care planning, (2) decision‐making and (3) development regarding care planning and decision‐making. The interviewers tried to minimise the strain on the fragile palliative patients in the interview situations and assisted them in recognising their care planning and decision‐making experiences. For example, instead of the concept of ACP, the concept of care planning was used because “ACP” is not an established concept in Finland. The interviews were carried out in face‐to‐face meetings in accordance with the wishes of the patients in hospital patient rooms. Only the interviewer and the patient were present. The interviews lasted between 9.30–50 min (mean 23 min), depending on the patient's condition. The interviewees gave permission for the interviews to be audio‐recorded. For most interviewees, a short appointment was the only possibility to conduct the interviews; it was not possible to continue them or take them up later. The data were considered sufficient and saturated when new cases no longer brought new information regarding the research question (Saunders et al. 2018).

**TABLE 1:** Interview guide.

Patient's experience of planning their own care
Theme 1. Care planning How has the care of your illness been planned? Could you give some examples? How have you taken part in planning your own care? When discussing your care with physicians and nurses ○ What aspects of your care have been planned? (CONTENT) ○ Have the objectives of your care been discussed? (objective) ○ Have you told them how you want to be cared for? (RESTRICTING CARE) ○ Have your wishes been listened to? ○ Have your feelings been listened to? ○ Do you think that you have been able to influence your care? How important do you consider that you can influence your care? What are the aspects of your care that you have planned?
Theme 2. Care decision‐making How have you taken part in the decision‐making concerning your care? Who have made decisions about your care? Have your own wishes been taken into account?
Theme 3. Development In your opinion, how should the care be developed?

### 2.4 Data Analysis

The data were analysed through inductive content analysis (Renjith et al. 2021). First, the data were analysed by one researcher (AK) and after that, discussed within the team. The data were transcribed verbatim, after which they were read through several times. Then, the data were searched for statements with the same content regarding advance care planning and decision‐making defined as units of analysis, which were simplified and coded with different colours. After that, simplifications that mean the same were combined as subcategories, which were given a name descriptive of the content. The analysis was continued by combining the subcategories with the same content into parent categories, whose correspondence to the original date was compared to contribute to rigorous data analysis. Lastly, parent categories with the same content were further combined and named main categories.

### 2.5 Ethical Considerations

This study complied with good scientific practice (World Medical Association 2024), with ethical approval (15/2019) and research permits obtained from the organisations. No relationship between interviewers and interviewees was established because the organisations were responsible for recruiting suitable and voluntary interviewees. Each ward had a contact person coordinating the study, who was responsible for the recruitment of the interviewees and for the distribution of the study information sheet and consent form. The interviewees were informed about the voluntary nature of participation and the right to withdraw. If the patient wanted to participate in the study, s/he was asked to express it by filling out the consent form and giving it to the contact person in a sealed envelope. The two researchers agreed on the time and place of the interview. Before the interview, interviewees were instructed about the course of the study. All interviewees filled out a background information form and gave their written consent. The interviewees consisted of a vulnerable patient group, and protecting them was of the utmost importance (Renjith et al. 2021). Thus, the researcher observed the interviewees' mental and physical resources and conditions during the interviews. If symptoms indicating deterioration of the physical condition had been observed, the interview would have been interrupted, and nursing staff would have been called to the scene. Moreover, the presence of a patient's family member at the time of the interview could have been comforting for some patients, and if necessary, it would have been offered.

## 3 Results

### 3.1 Description of the Interviewees

A total of 20 patients with cancer were interviewed. The patients were, on average, 75 years old (range 58–94 years). They had been suffering from cancer for an average of 3 years (< 0–17 years) and had been treated in a hospital palliative care ward for an average of 3 weeks (1–8 weeks).

### 3.2 Patients Experiences With Advance Care Planning in Palliative Care

Patients' experiences with advance care planning in palliative care were grouped into three parent categories: (1) making plans for the end of life, (2) symptom management planning, and (3) palliative care coordination (Table 2).

**TABLE 2:** Patients' experiences with advance care planning in palliative care.

Parent category	Subcategory
Making plans for the end of life	Need for psychosocial support in cancer disease
	Wish for goals of care discussion
Symptom management planning	Wish for pharmacological interventions for symptom management
	Wish for non‐pharmacological interventions for symptom management
Palliative care coordination	Need for discharge planning
	Wish for compatibility between team members

#### 3.2.1 Making Plans for the End of Life

The need for psychosocial support in cancer disease appeared in patients' experiences in different phases of cancer disease; in other words, in the diagnosis phase of a cancer disease, in connection with stopping active cancer treatments, and during the transition to follow‐up care. Patients in palliative care experienced that they and their family members were left on their own when they received the information about a fatal cancer diagnosis. Patients described that they had not received the support that they would have liked from healthcare professionals, e.g., in dealing with serious diagnostic information. Moreover, patients revealed gaps in continuity of care when their oncology treatments in the hospital had been stopped. Patients described that they and their family members were unsure of where they could get help. Finally, according to one patient (she), they had sought help on their own from a health care centre in primary care, where the patient had had a satisfying, several years‐long treatment relationship with the same healthcare professional, who had helped them cope with challenging situations. Patients described that they could have benefitted from peer support, i.e., meeting a similar patient with cancer. Patients had asked many times for such a person (patient), but they had been told that no such persons were available. On the other hand, according to the patients, the calm atmosphere in the palliative care ward, spirituality, and knowledge about the continuation of modern oncology treatments brought comfort and hope to them. In addition, patients hoped for family closeness, especially in the event of approaching death.

That (s)he has been there for all these years; we've had a good relationship (Interviewee 18).

Yes, I would have benefitted from someone who had the same… a similar case (Interviewee 7).

Wish for goals of care discussion meant that the patients expressed a desire for a discussion about how their treatment and care would continue. Interviewees experienced uncertainty about their future care scheme and schedule and described unawareness of their treatment goals. According to the patients, their follow‐up care plans were often unclear, incomplete, or completely missing. As an exception, one patient said that their care plan had been updated. Patients described that although they were in the palliative care ward, they had not necessarily yet been involved in the goals of care discussion with a physician, where they would have been listened to, and their current situation and possible outcomes of future treatment would have been reviewed. One patient described being in such poor health that they did not have the strength to think about end‐of‐life issues yet. They explained that they had been gathering strength and would then ask about everything that was bothering them if they had a discussion in the future. Patients described the melancholy that they felt being long‐term patients, and they felt that they could not have any great expectations in anticipation of the coming death. However, some patients still described that they received cytostatic treatment. Those patients waited for a consultation with the oncologist because, according to them, the continuation of their oncology treatment depended on the test results (e.g., concentration of cytostatics).

… someone like me, of course; now that I'm here, on a ward like this, it's pretty much like we haven't really… I think we could discuss this more or review the future (Interviewee 13).

At least up to now, I've not been anywhere where they would listen, talk about going forward, what's happening now, and what will be achieved if we try. In my opinion, I'm a long‐term patient, which means that for me, immediate care, that's death, it's written there in some documents. (Interviewee 4).

#### 3.2.2 Symptom Management Planning

Wish for pharmacological interventions for symptom management appeared according to patients in daily discussions between them and healthcare professionals, especially regarding medication. Patients described how they participated in planning pharmacological interventions related to managing their own symptoms of disease. For example, they spoke to nurses about their information needs, wishes, and fears regarding aspects such as pain management and prevention of epileptic seizures. In practice, they had spoken to nurses about where they had pain, what the pain was related to, or if they felt they needed more effective pain relief for night pains, for example. According to the patients, their information needs regarding symptomatic medication mainly concerned sedation and epileptic seizures. Patients described their wishes for sedation, especially if their pain had increased dramatically. In the patients' descriptions, their information needs concerning sedation consisted of how sedation is given, what follows from it, how it is monitored, and whether it can be implemented at different levels. In the patients' descriptions, knowledge of the possibility of receiving sufficient pain medication created a sense of security, but on the other hand, it also created a fear of losing consciousness. Patients described that they had received sufficient information to meet their information needs regarding the effects of their new epilepsy medication, particularly from the nurses.

Well, this is the only thing I'd like to … about sedation, like what's available, how it's given, what kind of signs there are, and so on. How it is monitored; Yes, how it is monitored, and can it be given at different levels? (Interviewee 7).

Yes, it does make me feel safe, but then again, I'm a bit frightened as well, like last night when I had visitors, and I really had this feeling that I don't want to be like this, in between, as it were. (Interviewee 7).

Wish for non‐pharmacological interventions for symptom management meant from the patient's point of view that the symptoms of their disease, such as pain, loss of appetite, vertigo, and muscle weakness, were planned to be treated with treatments other than medicines, and those interventions were included in their care plan. Patients illuminated elements of the content of planned interventions of their care. In accordance with the ideology of palliative care, according to the patients, the aim of planning the treatment of the symptoms of the disease was to make them feel as good as possible and to avoid suffering. A patient suffering from pain described it as a great thing to have their own space in the palliative care ward if necessary. One patient described that they did not want resuscitation because it would be unnecessary and increase the pain. Another patient suffering from loss of appetite described that/they had eaten decent portions because they had been served the food they wanted in the palliative care ward. Patients also described taking care of the mobility exercises independently or waiting to get out with their family members.

Well, not really, the situation being what it is, and, well, the result is that actually… the aim here is for me to feel as good, good—well, as good as possible. (Interviewee 3).

It has spread so much that just to be rid of the pain … I don't think there is much else (Interviewee 16).

#### 3.2.3 Palliative Care Coordination

Need for discharge planning appeared according to patients in taking care of continuity of palliative care, especially when arranging their follow‐up care after the hospital period. The patients in palliative care described that safe discharge from hospital to home included arrangement of necessary support such as home care services or hospital‐at‐home services as well as family member involvement. Moreover, discharge planning from the hospital ward involved preparing various assistive tools and devices, such as a rollator or a security phone at their home.

Patients considered it very important that once at home after the hospital period, they had a known contact channel for any emergency situations. Patients expressed satisfaction with the fact that they always had a place in the palliative care ward where they could return at any time if necessary. On the other hand, some palliative patients described a break in the continuity of palliative care when they had not been able to return directly to the palliative care ward from home care services, even though they had been promised that and 24/7 help. They described unpleasant experiences when they had to go back to the palliative care ward through the emergency department (ED) in poor condition.

When the doctor said that you can come back at any time, you can just come in; there is a place for me at all times. That is truly a wonderful feeling. (Interviewee 20).

I really cannot say anything nice about emergency departments; they are horrible—have you been to one yourself? (Interviewee 20).

Wish for compatibility between team members meant to patients the significance of compatibility with the wishes of their own family members and healthcare professionals in palliative care. Patients described that a suitable end‐of‐life setting was very important for them because they assessed they might require end‐of‐life care for a long time. One patient said that moving from one place to another would be a disaster for them now, while another patient was of the opinion that the palliative care ward was perceived as a temporary place of care from which the end‐of‐life care setting was organised. According to the patients, the involvement of family members in end‐of‐life care setting planning was considered important. Patients described that their family members had, e.g., already been involved in end‐of‐life care setting planning, such as determining where the patient was transferred after the hospital period. The patients who assessed that the palliative care ward was the best place for them described worries about the costs and how long they could stay there. Especially patients living alone said they were afraid of being discharged home from the hospital. Patients who had experiences of hospice said that they valued the well‐functioning services of the hospice, the sense of community and, on the other hand, the opportunity for privacy there. Patients described sharing responsibility for the development of the ward's functions but were unsure of their own influence and saw that society was responsible for organising a home‐like end‐of‐life care setting. According to the patients, hospices should definitely remain in place and not be closed down under any circumstances, as was being done at the time of the interviews.

Well, like I said, I'm really scared of what it's like at home, as all kinds of things can happen (Interviewee 14).

…so this system is something that society needs to provide, like there has to be a home‐like place that you can go to. (Interviewee 4).

### 3.3 Patients' Experiences of With Care Decision‐Making in Palliative Care

Patients' experiences with care decision‐making in palliative care were grouped into two parent categories: (1) healthcare professional as a care decision‐maker and (2) shared decision‐making (Table 3).

**TABLE 3:** Patients' experiences with care decision‐making in palliative care.

Parent category	Subcategory
Healthcare professional as a care decision‐maker	Medical care decision‐making
	Nursing care decision‐making
	Interprofessional care decision‐making
Shared decision‐making	Need for patient involvement in shared decision‐making
	Need for family member involvement in shared decision‐making

#### 3.3.1 Healthcare Professional as a Care Decision‐Maker

Medical care decision‐making meant decisions made by physicians. According to patients' experiences, medical decision‐making included, for example, decisions regarding treatment of cancer disease or patient transfers between different treatment facilities. Patients described having confidence in physicians' expertise in disease treatment planning, such as the planning of oncological treatments, but on the other hand, some patients felt they had to settle for the decisions made by physicians. Patients described confidence that physicians knew best because they were experts in medical issues. Similarly, some patients experienced that they did not want to say how they wanted to be treated. The patients described that from their point of view, in the early stages of cancer disease, it is difficult for the patient to take a stand on oncological treatment planning. Correspondingly, a patient who had undergone hard oncology treatment periods described that the treatment decisions regarding cancer disease were made and came “from above”, without discussing them with the patient. An informant suffering from severe pain symptoms described their experience of submitting to medical decisions in a situation where they had been transferred from one treatment facility to another in great pain against their will, without their opinion being heard and taken into account.

…so there is really nothing this so‐called patient can say about it; it all comes from higher up, as it were. And you just have to accept it… (Interviewee 4).

…and for me, the pain was already really bad there at X, so I wasn't ready to go anywhere. (Interviewee 7).

Nursing care decision‐making meant decisions made by nurses. According to the patients' experiences, nursing care decision‐making included, for example, decisions regarding daily nursing care with nursing interventions. Patients described that nurses took care of and made decisions on issues such as those related to their wound care or bowel function based on their professional qualifications. Patients said they were aware of the existence of their nursing records. That is why they hoped that nursing staff would also base their nursing care decisions on their documented patient‐specific care needs. Sometimes, the patients had noticed that the nursing staff may not continuously be familiarised with their care plan information before performing nursing interventions. For example, one patient suffering from vertigo described information gaps related to nursing care planning information because the patient's non‐pharmacological intervention for the treatment of vertigo was not registered in the palliative ward. The patient had passed out because the information in their patient records about food with extra salt and Vichy for vertigo had, for some reason, not been registered or noted. For this reason, the patient had to point this out to the nurses. Thus, according to patients, nurses should familiarise themselves more specifically with patients' nursing reports in the Nursing Documentation Systems (NDS) and utilise the recorded information as a basis for treatment decisions.

…and in my case, my bowels need to function, so I have to eat that powder (Interviewee 20).

Well it does seem a bit like they don't read them properly; you need to point things out at times (Interviewee 8).

Inter‐professional care decision‐making meant decisions made jointly by nurses and physicians. According to the patients' experience, inter‐professional care decision‐making at the cancer care clinic included, for example, planning the schedule for a given day by the healthcare team. Patients described that general (treatment and care) instructions came from physicians and nursing staff. That is why some patients even assessed their own influence on care decision‐making as low.

So, they like made the decisions together there. (Interviewee 1).

…for the patient, well, it comes from the doctor and the staff; that's where it comes from. So, there is not that much you can do about it yourself. (Interviewee 4).

#### 3.3.2 Shared Decision‐Making

Need for patient involvement in shared decision‐making meant that nurses and physicians asked their opinions and considered them in care planning and decision‐making. Patients reported and appreciated that they had had an opportunity to express their opinions and thus participate in decisions regarding their own treatment, for example, when planning different treatment methods, the place of further care in transition situations, and whenever necessary. However, according to patients, their involvement in decision‐making was not self‐evident. Sometimes it depended on their own activity; otherwise, they would have been excluded from SDM. Patients described that sometimes, the physician did not seem to know enough about the modern oncology treatment that the patient was receiving to slow down the progression of cancer. In such cases, they had to search for additional information themselves.

However, it's quite nice, that ‘should be start off with this now’ (Interviewee 1).

The need for family member involvement in shared decision‐making meant that nurses and physicians asked for family members' opinions and took them into account in care planning and decision‐making. The patients described that their family members were aware of the progress of their treatment schedule, acted as their guardians and, if necessary, would make (end‐of‐life) care decisions in accordance with their wishes. The patients provided practical examples of this. Their family members had, for example, participated in medical rounds in the hospital palliative care ward or had brought literature about modern oncology treatment from abroad to support SDM. Some patients described that they had discussed their end‐of‐life wishes with their family members. Those patients had empowered their family members to participate in SDM about their future care through a living will. In the living will, they had expressed their refusal to be resuscitated. The patients described that their family members were the most important advocates for them, and they trusted their family members to make decisions according to their wishes based on facts and the patient's wishes. Finally, patients brought up their concerns about those patients who did not have an advocate in the constantly changing social and healthcare service system.

The children know and decide, so there won't be any resuscitation or things like that; we have gone through all that. (Interviewee 5).

Yes, I can see that those who have no one to advocate for them; I feel sorry for them. (Interviewee 5).

## 4 Discussion

The study findings describe patients' experiences with advance care planning and decision‐making in palliative care.

In this study, patient experiences with advance care planning in palliative care were described in relation to making plans for the end of life, symptom management planning and palliative care coordination. Patients in palliative care wards experienced high mental stress and reported a great need for psychosocial support for coping at the seams of the continuum of care in different phases of cancer disease. Patients were looking forward to having goals of care discussions earlier in the illness trajectory to meet their preferences. Based on Finnish law (Act on the Status and Rights of Patients 1992), patients have a statutory right to access information about their treatment and also the right to a care plan, which is drawn up together with a healthcare professional. From the perspective of preventive healthcare and patients' legal right to access information, it is worrying that the flow of information between staff and patients is far from optimal. Important information that patients should self‐evidently have access to has not been given to them. Possible Finnish cultural influences that make it hard to talk about death may have affected the participants. Resources related to the patient's mental well‐being and physical health are limited at the end of life, and they do not have time for a long therapy process, for example. Previously, psychosocial support has been provided successfully to patients with cancer with the aid of ACP. According to an integrative literature review, the positive outcomes of the use of ACP have included a reduction in patients' anxiety and depression. (Goswami 2023).

Many patients in this study had a desire for a discussion, especially with a physician. In general, patients are willing to share their end‐of‐life care wishes but are waiting for someone to speak up (van Doorne et al. 2021). According to a systematic review, the obstacles to the ACP discussion are more generally professional than patient‐derived (Guccione et al. 2023). Earlier scoping review evidence supports the fact that besides medical professionals, also nursing professionals can be involved in the preparation of ACP (Kuusisto et al. 2020). For a nurse who is available in the ward 24/7, this can be easier than for physicians when the nurse notices a patient's readiness or a change in their condition. According to a study in the USA, a brief motivational interview intervention by a nurse to stimulate conversation about serious illness was shown to increase self‐reported ACPs in those patients who were at high risk of short‐term mortality (Ouchi et al. 2023). Moreover, a patient who has experienced the same things can provide psychosocial support and give hope because s/he already has the knowledge and experience (Kowitt et al. 2019).

This study identified a need for a standardised digital palliative service pathway to support integrated palliative care and cancer care coordination (outpatient, inpatient, and community‐based settings) proposed by the Lancet Oncology Commission, where patients' treatment is based on a treatment plan according to their treatment needs (Kaasa et al. 2018). Similarly to this study, in Denmark, patients with cancer disease in their last stage of life were afraid of symptoms and of going to the ED and were hoping to control the symptoms (pain) for which they had attended the ED (van der Padt‐Pruijsten et al. 2022). A functioning palliative care service chain is thus essential because palliative care without a care chain does not allow for the continuity of care that patients expect and desire. Patients should always be aware of the right information channel after hospital discharge and who to contact if necessary. If possible, similarly to the study of Hui et al. (2023), telemedicine appointments could be used for symptom management and goals‐of‐care discussions. Moreover, according to Bukstein et al. (2020), shared decision support systems should be integrated into professionals' workflows; through that, trust can be built between patients and professionals.

The patients in this study brought up the importance of their individual needs and of coordinating the wishes of different parties, especially their families, in end‐of‐life care setting planning. According to the patients, their own home was not the only optimal treatment place; the palliative care ward and the hospice were also options. In a previous study, the process of draughting end‐of‐life setting preferences depended on many things and was shaped by uncertainty related to the illness, family, and services (Gerber et al. 2019). An intervention study in the Netherlands has shown that the use of the structured advance care planning tool (ACPT) in end‐of‐life care planning has reduced the use of health care services, and patients have died in a place of their choice (Burghout et al. 2023).

In this study, patients' experiences with care decision‐making in palliative care were described in relation to healthcare professionals as a care decision‐maker and shared decision‐making. According to patients, care decision‐making was largely driven by the healthcare team, which affected participants' perception of the “planned” quality of interventions. Trust in healthcare professionals' expertise in medical care treatment planning was highlighted by many patients, but some experienced that their medical care interventions had been given as a dictation policy, without taking into account their own opinions, or even wanting to ask anything “about” their cancer disease. Similarly, a previous study in Sweden revealed that according to nurses and physicians, end‐of‐life care planning was medical‐focused at the expense of the patient's psychosocial care planning needs, which limited patient‐centred decision‐making guidance (Beck et al. 2023). However, according to a systematic review and meta‐analysis, ACP is a feasible and effective strategy to make it easier for patients to express their healthcare wishes in a timely manner and to improve their outcomes (Wang et al. 2023). In this study, a few patients with cancer mentioned that they were being treated with new, modern oncology treatments, which gave them hope for the future. Recently, Temel et al. (2022) presented that patients who receive the aforementioned new individualised oncology treatments near end‐of‐life care have not necessarily participated in end‐of‐life discussions in time. Moreover, these new oncological treatments have brought uncertainty to patients' prognosis and created new challenges for professionals to take into account patients' autonomy in end‐of‐life care planning with ACP.

This study highlights the synergy between advance care planning and decision‐making and the importance of patient health records and their utilisation to enable patient‐centred care. The potential absence of nursing notes and interruptions in the transfer of information hinder the continuity of care and interfere with the realisation of patient‐centred care. Previously in Sweden, a retrospective literature review verified that patient‐specific information regarding treatment of palliative patients had not been found in the electronic health record because there were no nursing care plans and the information in question had not been recorded in a structured way (Sjöberg et al. 2022).

In this study, shared decision‐making appeared to be patient‐specific when patients and family members had been involved in their palliative care planning and in decisions about their care. The patients in this study brought up the central role of their family members in care decision‐making about their care. Today, different shared decision‐making support systems and aids can be integrated into healthcare professionals' workflows. They enable healthcare professionals to understand (using displayed prompts) patients' values and thoughts, and through this, important trust can be built between patients, family members and professionals (Bukstein et al. 2020). According to a systematic review, technology‐enabled delivery modes, consultations, structured meetings and printed material were previously used interventions to support shared decision‐making in palliative care (Baik et al. 2019). In the USA, Volandes et al. (2023) showed with an experimental study design that with the use of an evidence‐based video‐assisted decision‐making support tool, nurses and social workers were able to facilitate Shared Decision‐Making discussions, and recorded goals‐of‐care discussions increased compared to the traditional procedure.

### 4.1 Strengths and Limitations

It should be taken into account that all participants in this study were Finns by ethnic background and receiving palliative care in a hospital ward setting. Palliative care is also offered in hospice care or at home, and those persons were not represented in this study. Due to the nature of these different services, there can be very different experiences with advance care planning and SDM than in a more structured hospital ward. However, some study participants described their experiences during cancer treatment trajectory in general.

All the interviewees had cancer disease, even though the study inclusion criteria did not limit the interviewees only to that patient group. Patients who were in poor physical condition, confused, or anxious were deemed unsuitable to participate in the study. No transcripts could be returned to participants for comment and/or corrections due to the deteriorating condition of the patients. To ensure the consistency of the data collection by two researchers, the interviewers used an interview guide (Table 1), the uniform use of which had been instructed in advance. The researchers' experience in palliative care and its possible influence on the interpretation of the data were identified at the beginning of the research process. For this reason, throughout the research process, objectivity was strengthened, and possible biases were consciously set aside so that the data would directly reflect the patients' experiences. (Renjith et al. 2021). Finally, one author (AK) conducted the data analysis, but the research team discussed and checked the results to ensure confirmability and trustworthiness.

## 5 Conclusion

This study highlights the need for advance care planning to be involved in shared decision‐making in palliative care from patients' perspectives. The results from this study show that early and intentional initiation of palliative care is of critical concern for nurses. In the future, comparing patients' and healthcare professionals' experiences of advance care planning and decision‐making could warrant further research.

## Author Contributions

Study concept and design: A.K., E.H. Data analysis and interpretation: A.K., K.S., E.H. Draughting of the article: A.K. Critical revision of the article: A.K., A.S.‐J., E.H., K.L., K.S.

## Conflicts of Interest

The authors declare no conflicts of interest.

## Supporting information


Data S1.

## Data Availability

Data not available due to ethical restrictions.

## References

[nop270158-bib-0001] Act on the Status and Rights of Patients . 1992. https://www.finlex.fi/en/laki/kaannokset/1992/en19920785_20120690.pdf. 11651575

[nop270158-bib-0002] Baik, D. , H. Cho , and R. M. Masterson Creber . 2019. “Examining Interventions Designed to Support Shared Decision Making and Subsequent Patient Outcomes in Palliative Care: A Systematic Review of the Literature.” American Journal of Hospice & Palliative Care 36, no. 1: 76–88. 10.1177/1049909118783688. 29925244 PMC6056336

[nop270158-bib-0003] Beck, S. , L. Lundblad , C. Göras , and M. Eneslätt . 2023. “Implementing Advance Care Planning in Swedish Healthcare Settings–A Qualitative Study of Professionals' Experiences.” Scandinavian Journal of Primary Health Care 41, no. 1: 23–32. 10.1080/02813432.2022.2155456. 36519794 PMC10088918

[nop270158-bib-0004] Bukstein, D. A. , D. G. Guerra Jr , T. Huwe , and R. A. Davis . 2020. “A Review of Shared Decision‐Making: A Call to Arms for Health Care Professionals.” Annals of Allergy, Asthma & Immunology 125, no. 3: 273–279. 10.1016/j.anai.2020.06.030. 32603786

[nop270158-bib-0005] Burghout, C. , L. M. V. Nahar‐van Venrooij , S. R. Bolt , T. J. Smilde , and E. J. M. Wouters . 2023. “Benefits of Structured Advance Care Plan in End‐of‐Life Care Planning Among Older Oncology Patients: A Retrospective Pilot Study.” Journal of Palliative Care 38, no. 1: 30–40. 10.1177/08258597221119660. 36039518

[nop270158-bib-0006] Coulter, A. , V. A. Entwistle , A. Eccles , S. Ryan , S. Shepperd , and R. Perera . 2015. “Personalised Care Planning for Adults With Chronic or Long‐Term Health Conditions.” Cochrane Database of Systematic Reviews 2015, no. 3: CD010523. 10.1002/14651858.CD010523. 25733495 PMC6486144

[nop270158-bib-0007] Ellis, E. M. , E. Orehek , and R. A. Ferrer . 2019. “Patient‐Provider Care Goal Concordance: Implications for Palliative Care Decisions.” Psychology & Health 34, no. 8: 983–998. 10.1080/08870446.2019.1584672. 30905185

[nop270158-bib-0008] Gerber, K. , B. Hayes , and C. Bryant . 2019. “‘It All Depends!’: A Qualitative Study of Preferences for Place of Care and Place of Death in Terminally Ill Patients and Their Family Caregivers.” Palliative Medicine 33, no. 7: 802–811. 10.1177/0269216319845794. 31046580

[nop270158-bib-0009] Goswami, P. 2023. “Impact of Advance Care Planning and End‐of‐Life Conversations on Patients With Cancer: An Integrative Review of Literature.” Journal of Nursing Scholarship 55, no. 1: 272–290. 10.1111/jnu.12804. 35946931

[nop270158-bib-0010] Guccione, L. , S. Fullerton , K. Gough , et al. 2023. “Why Is Advance Care Planning Underused in Oncology Settings? A Systematic Overview of Reviews to Identify the Benefits, Barriers, Enablers, and Interventions to Improve Uptake.” Frontiers in Oncology 13: 1040589. 10.3389/fonc.2023.1040589. 37188202 PMC10175822

[nop270158-bib-0011] Hui, D. , B. Sakamoto Ribeiro Paiva , and C. E. Paiva . 2023. “Personalizing the Setting of Palliative Care Delivery for Patients With Advanced Cancer: ‘Care Anywhere, Anytime’.” Current Treatment Options in Oncology 24, no. 1: 1–11. 10.1007/s11864-022-01044-1. 36576706 PMC9795143

[nop270158-bib-0012] Institute of Medicine . 2014. “Dying in America. Summary.” Study Findings and Recommendations. https://www.ncbi.nlm.nih.gov/books/NBK285681/.

[nop270158-bib-0013] Kaasa, S. , J. H. Loge , M. Aapro , et al. 2018. “Integration of Oncology and Palliative Care: A Lancet Oncology Commission.” Lancet Oncology 19, no. 11: e588–e653. 10.1016/S1470-2045(18)30415-7. 30344075

[nop270158-bib-0014] Kim, S. , A. Lim , H. Jang , and M. Jeon . 2023. “Life‐Sustaining Treatment Decision in Palliative Care Based on Electronic Health Records Analysis.” Journal of Clinical Nursing 32, no. 1–2: 163–173. 10.1111/jocn.16206. 35023248 PMC10078701

[nop270158-bib-0015] Kowitt, S. D. , K. R. Ellis , V. Carlisle , et al. 2019. “Peer Support Opportunities Across the Cancer Care Continuum: A Systematic Scoping Review of Recent Peer‐Reviewed Literature.” Supportive Care in Cancer 27, no. 1: 97–108. 10.1007/s00520-018-4479-4. 30293093 PMC6561346

[nop270158-bib-0016] Kuosmanen, L. , M. Hupli , S. Ahtiluoto , and E. Haavisto . 2021. “Patient Participation in Shared Decision‐Making in Palliative Care–An Integrative Review.” Journal of Clinical Nursing 23–24: 341–3428. 10.1111/jocn.15866. 34028923

[nop270158-bib-0036] Kuusisto, A. , J. Santavirta , K. Saranto , P. Korhonen , and E. Haavisto . 2020. “Advance Care Planning for Patients with Cancer in Palliative Care: A Scoping Review from a Professional Perspective.” Journal of Clinical Nursing 29, no. 13–14: 2069–2082. 10.1111/jocn.15216. 32045048

[nop270158-bib-0035] Kuusisto, A. , K. Saranto , P. Korhonen , and E. Haavisto . 2023. “End‐of‐Life Discussions from the Perspective of Social Care and Healthcare Professionals in Palliative Care.” Omega Westpoint: 302228231185172. 10.1177/00302228231185172. PMC12444026 37342869

[nop270158-bib-0017] Ouchi, K. , R. S. Lee , S. D. Block , et al. 2023. “An Emergency Department Nurse Led Intervention to Facilitate Serious Illness Conversations Among Seriously Ill Older Adults: A Feasibility Study.” Palliative Medicine 37, no. 5: 730–739. 10.1177/02692163221136641. 36380515 PMC10183478

[nop270158-bib-0018] Queensland Health . 2019. “Care Plan for the Dying Person. Health Professional Guidelines.” Part 1: Overview of the CPDP. https://www.health.qld.gov.au/__data/assets/pdf_file/0023/833315/cpdp‐care‐plan‐hp‐guidelines.pdf.

[nop270158-bib-0019] Renjith, V. , R. Yesodharan , J. A. Noronha , E. Ladd , and A. George . 2021. “Qualitative Methods in Health Care Research.” International Journal of Preventive Medicine 12: 20. 10.4103/ijpvm.IJPVM_321_19. 34084317 PMC8106287

[nop270158-bib-0020] Rietjens, J. A. C. , R. L. Sudore , M. Connolly , et al. 2017. “Definition and Recommendations for Advance Care Planning: An International Consensus Supported by the European Association for Palliative Care.” Lancet Oncology 18: e543–551. 28884703 10.1016/S1470-2045(17)30582-X

[nop270158-bib-0021] Saunders, B. , J. Sim , T. Kingstone , et al. 2018. “Saturation in Qualitative Research: Exploring Its Conceptualization and Operationalization.” Quality & Quantity 52, no. 4: 1893–1907. 10.1007/s11135-017-0574-8. 29937585 PMC5993836

[nop270158-bib-0022] Sharafi, S. , A. Ziaee , and H. Dahmardeh . 2022. “What Are the Outcomes of Hospice Care for Cancer Patients? A Systematic Review.” Supportive Care in Cancer 31, no. 1: 64. 10.1007/s00520-022-07524-2. 36538122

[nop270158-bib-0023] Sjöberg, M. , B. H. Rasmussen , A.‐K. Edberg , and I. Beck . 2022. “Existential Aspects Documented in Older People's Patient Records in the Context of Specialized Palliative Care: A Retrospective Review.” BMC Health Services Research 22, no. 1: 1356. 10.1186/s12913-022-08753-1. 36384554 PMC9667671

[nop270158-bib-0024] Staats, K. , B. Ervik , and S. E. Fæø . 2023. “The Feeling of Being Home When Nearing End‐of‐Life—The Example of Norway: A Discussion Paper.” Nordic Journal of Nursing Research 43, no. 2: 1–4. 10.1177/20571585231180185.

[nop270158-bib-0025] Starr, L. T. , C. M. Ulrich , K. L. Corey , and S. H. Meghani . 2019. “End‐of‐Life Discussions, Health‐Care Utilization, and Costs in Persons With Advanced Cancer: A Systematic Review.” American Journal of Hospice & Palliative Care 36, no. 10: 913–926. 10.1177/1049909119848148. 31072109 PMC6711813

[nop270158-bib-0026] Temel, J. S. , L. A. Petrillo , and J. A. Greer . 2022. “Patient‐Centered Palliative Care for Patients With Advanced Lung Cancer.” Journal of Clinical Oncology 40, no. 6: 626–634. 10.1200/JCO.21.01710. 34985932

[nop270158-bib-0027] Tong, A. , P. Sainsbury , and J. Craig . 2007. “Consolidated Criteria for Reporting Qualitative Research (COREQ): A 32‐Item Checklist for Interviews and Focus Groups.” International Journal for Quality in Health Care: Journal of the International for Society for Quality in Health Care 19: 349–357. 10.1093/intqhc/mzm042 17872937

[nop270158-bib-0028] van der Padt‐Pruijsten, A. , T. Oostergo , M. B. L. Leys , C. C. D. van der Rijt , and A. van der Heide . 2022. “Hospitalisations of Patients With Cancer in the Last Stage of Life. Reason to Improve Advance Care Planning?” European Journal of Cancer Care 31, no. 6: e13720. 10.1111/ecc.13720. 36172990 PMC9788226

[nop270158-bib-0029] van Doorne, I. , M. van Rijn , S. M. Dofferhoff , D. L. Willems , and B. M. Buurman . 2021. “Randomized Controlled Trial.” Age and Ageing 50, no. 6: 2004–2011. 10.1093/ageing/afab176. 34473834 PMC8581384

[nop270158-bib-0030] Volandes, A. E. , S. N. Zupanc , J. R. Lakin , et al. 2023. “Video Intervention and Goals‐Of‐Care Documentation in Hospitalized Older Adults: The VIDEO‐PCE Randomized Clinical Trial.” JAMA Network Open 6, no. 9: e2332556. 10.1001/jamanetworkopen.2023.32556. 37695586 PMC10495866

[nop270158-bib-0031] Wang, X. , X.‐L. Huang , W.‐J. Wang , and L. Liao . 2023. “Advance Care Planning for Frail Elderly: Are We Missing a Golden Opportunity? A Mixed‐Method Systematic Review and Meta‐Analysis.” BMJ Open 13, no. 5: e068130. 10.1136/bmjopen-2022-068130. PMC10230943 37247960

[nop270158-bib-0032] World Medical Association . 2024. “WMA Declaration of Helsinki—Ethical Principles for Medical Research Involving Human Subjects.” https://www.wma.net/policies‐post/wma‐declaration‐of‐helsinki‐ethical‐principles‐for‐medical‐research‐involving‐human‐subjects/. 19886379

[nop270158-bib-0033] Zhu, N. , L. Yang , X. Wang , et al. 2023. “Experiences and Perspectives of Healthcare Professionals Implementing Advance Care Planning for People Suffering From Life‐Limiting Illness: A Systematic Review and Meta‐Synthesis of Qualitative Studies.” BMC Palliative Care 22, no. 1: 55. 10.1186/s12904-023-01176-7. 37149560 PMC10163819

[nop270158-bib-0034] Zulueta Egea, M. , M. Prieto‐Ursúa , L. Bermejo Toro , and A. M. Palmar‐Santos . 2023. “Dimensions of Good Palliative Nursing Care: Expert Panel Consensus and Perceptions of Palliative Professionals.” Journal of Clinical Nursing 32, no. 13–14: 3746–3756. 10.1111/jocn.16583. 36380458

